# Impact of lockdown on key workers: findings from the COVID-19 survey in four UK national longitudinal studies

**DOI:** 10.1136/jech-2020-215889

**Published:** 2021-04-09

**Authors:** Constantin-Cristian Topriceanu, Andrew Wong, James C Moon, Alun D Hughes, Nishi Chaturvedi, Gabriella Conti, David Bann, Praveetha Patalay, Gabriella Captur

**Affiliations:** 1 MRC Unit for Lifelong Health and Ageing, University College London, London, UK; 2 Institute of Cardiovascular Science, University College London, London, UK; 3 Cardiac MRI Unit, Barts Heart Center, London, UK; 4 Department of Economics and UCL Social Research Institute, University College London, London, UK; 5 Centre for Longitudinal Studies, Social Research Institute, University College London, London, UK; 6 Center for Inherited Heart Muscle Conditions, Royal Free Hospital, London, UK

**Keywords:** epidemiology, health impact assessment, public health

## Abstract

**Background:**

Key workers played a pivotal role during the national lockdown in the UK’s response to the COVID-19 pandemic. Although protective measures have been taken, the impact of the pandemic on key workers is yet to be fully elucidated.

**Methods:**

Participants were from four longitudinal age-homogeneous British cohorts (born in 2001, 1990, 1970 and 1958). A web-based survey provided outcome data during the first UK national lockdown (May 2020) on COVID-19 infection status, changes in financial situation, trust in government, conflict with people around, household composition, psychological distress, alcohol consumption, smoking and sleep duration. Generalised linear models with logit link assessed the association between being a key worker and the above outcomes. Adjustment was made for cohort design, non-response, sex, ethnicity, adult socioeconomic position (SEP), childhood SEP, the presence of a chronic illness and receipt of a shielding letter. Meta-analyses were performed across the cohorts.

**Findings:**

13 736 participants were included. During lockdown, being a key worker was associated with increased chances of being infected with COVID-19 (OR 1.43, 95% CI 1.22 to 1.68) and experiencing conflict with people around (OR 1.19, 95% CI 1.03 to 1.37). However, key workers were less likely to be worse off financially (OR 0.32, 95% CI 0.24 to 0.65), to consume more alcohol (OR 0.88, 95% CI 0.79 to 0.98) or to smoke more (OR 0.60, 95% CI 0.44 to 0.80) during lockdown. Interestingly, being a key worker was not associated with psychological distress (OR 0.95, 95% CI 0.85 to 1.05).

**Interpretation:**

Being a key worker during the first UK COVID-19 lockdown was a double-edged sword, with both benefits and downsides. The UK government had the basic duty to protect its key workers from SARS-CoV-2 infection, but it may have failed to do so, and there is an urgent need to rectify this in light of the ongoing third wave.

## Introduction

The WHO declared the SARS-CoV-2/COVID-19 outbreak a global pandemic on 11 March 2020. In an attempt to contain and limit the spread of the virus, the UK government imposed a national lockdown on 23 March 2020 across England, Scotland and Wales. The restrictions were gradually relaxed starting from June 2020.

Key worker occupations refer to those that are vital for the COVID-19 pandemic response. They account for 33% of the total national workforce according to the UK Office for National Statistics. Although the most common group of key worker occupations are health and social care workers, the majority of key workers actually work in non-health and social care sectors such as in education and childcare, key public services, transport and utilities.

During the UK national lockdown, key workers played a pivotal role in the COVID-19 response. Studies emerged showing that during the pandemic, key workers were suffering from negative health such as more COVID-19 infections^1^ and mental distress.^2 3^ Their labour has been praised by the media and there have been multiple editorial letters calling for their better support and protection.^1 4^ Various societal efforts such as the ‘clap for carers’ campaign, food vouchers, free food deliveries, free transportation and parking, and priority access at supermarkets were established during lockdown in an attempt to alleviate some of the burdens faced by key workers. However, the impact of the COVID-19 lockdown on key workers is yet to be fully elucidated.

To answer this question, we used electronic survey data captured at the peak of the UK national lockdown (May 2020) from participants of four UK longitudinal studies spanning multiple generations (18–62 years old). In this exploratory study, we sought to investigate whether being a key worker during lockdown was associated with a set of health and socioeconomic outcomes selected based on previous key worker findings,^1–4^ lockdown trends already observed for the general population (Bann *et al,* 2020) and topics of public interest as reported in the media.

## Methods

### Study design

Participants were from three UK birth cohorts: National Child Development Study (NCDS) born in 1958,^5^ 1970 British Cohort Study (BCS70) born in 1970^6^ and Millennium Cohort Study (MCS) born in 2000–2002^7^; and one national longitudinal study with follow-up from adolescence: Next Steps (NS) born in 1989–1990.^8^

During the UK national lockdown, all participants who were alive and not lost to follow-up were sent (between 2 May 2020 and 1 June 2020) an online questionnaire which measured demographic, behavioural and health variables. Survey questions used to derive the exposures, covariates and outcomes of this study are presented in online supplemental table S1. The full survey questions can be accessed here: (https://cls.ucl.ac.uk/wp-content/uploads/2020/12/COVID-19-Online-Survey-Questionnaire-Wave-1-April-2020-Version-2.pdf). Participants provided informed consent.

10.1136/jech-2020-215889.supp1 Supplementary data

### Outcomes

COVID-19 infection was recoded as 0=no and 1=yes, based on a positive antigen or antibody test or strong personal suspicion due to symptoms. Change in financial situation, trust in government and conflict during lockdown (compared with before the COVID-19 outbreak) were standalone self-rated survey question with three answer options: less/worse, same or more/better. We recoded the change in financial situation and trust in government as 0=same or more/better and 1=less/worse. The change in conflict with the people around was recoded as 0=less and 1=same or more. Respondents were also asked if there has been a change in household composition with 0=no and 1=yes available as options. We calculated change scores for psychological distress, alcohol, smoking and sleep. Mental health state was measured both before the outbreak and during the national lockdown using the General Health Questionnaire (GHQ-12) for NS,^9^ using a shortened 9-item Malaise inventory for NCDS and BCS70,^10 11^ and using the Kessler K6 score for MCS.^12^ Psychological distress was defined as an increase in the corresponding mental state score during compared with the pre-pandemic state. Alcohol consumption was captured as two variables: how often (0–≥4 times per week) and how many (number of drinks per day). A composite variable was computed for the number of drinks per week. An increase in the number of drinks per week during lockdown compared with the pre-pandemic state was scored as 1, and 0 otherwise. For smoking, we calculated the difference between the number of cigarettes smoked before and during lockdown, thus recoding as 0=same or fewer, and 1=more cigarettes smoked. Lastly, participants were asked how many hours of sleep they had per night on average during lockdown and pre-pandemic. Participants who slept less hours were scored as 1, while the remaining were scored as 0.

### Exposures

Key worker status was self-assigned based on whether the participant believed their work has been classified as critical to the COVID-19 response. Participants actively working during lockdown, but not as key workers, were referred to as other workers. Participants in unpaid employment or volunteers were referred to as volunteers. Participants who were not in paid or unpaid employment but were receiving teaching during lockdown were referred to as in teaching.

### Covariates

Sex was recoded as 0=male and 1=female, while ethnicity was recoded as 0=White and 1=non-White. NCDS and BCS70 have a very small proportion of non-white participants, thus, ethnicity differences were assessed only for the NS and MCS cohorts. Adult socioeconomic position (SEP) was defined according to the highest educational attainment: degree/higher, advanced-level examination/diploma, ordinary-level examination/General Certificate of Secondary Education or none. For MCS participants who were still in education, their parents’ highest education attainment was used instead. Childhood social class has been recorded according to the UK Office of Population Censuses and Surveys Registrar General’s social class as: professional, managerial and technical, skilled non-manual, skilled manual, partly skilled and unskilled. The presence (yes/no) and nature of any long-standing chronic illnesses were also noted. Individuals who were considered to be at high risk of negative health outcomes in the event of COVID-19 infection should have received a shielding letter, so receipt of a shielding letter was also recorded (yes/no).

### Statistical analysis

Statistical analysis was performed in R (V.3.6.3). Histograms enabled visual assessment of data. Categorical variables were expressed as counts and per cent for each available category.

Weights to account for the stratified designs of NS and MCS cohorts have been previously developed. Logistic regression models predicting the response during the COVID-19 data sweep based on demographic, socioeconomic, household and individual predictors of non-response at previous data collection points were used to calculate non-response weights. In the logistic regression models, missing covariate values were generated using multiple imputation. For the COVID-19 survey respondents, the probability of response was predicted, and non-response weights were derived as the inverse of the probability of response and were further calibrated so they sum to the number of respondents in each cohort. The stratified survey design and non-response weights were combined to generate an individualised combined weight for each study respondent (full details available here in the Centre for Longitudinal Study COVID-19 Survey User Guide: https://cls.ucl.ac.uk/wp-content/uploads/2020/12/UCL-Cohorts-COVID-19-Survey-user-guide.pdf).

Generalised linear regression models with logit link were developed to assess whether key worker status was associated with COVID-19 infection; changes in financial situation; trust in government; conflict; household composition; psychological distress; alcohol consumption; smoking and sleep duration during lockdown. For each outcome, the regression models were adjusted for the combined weight, sex, ethnicity, adult SEP, childhood SEP, the presence of a chronic illness and the receipt of a shielding letter. Each analysis was initially conducted per cohort. Meta-analyses were then performed across the cohorts for each outcome. Cochran’s Q p value and Higgins I^2^ were employed to assess study heterogeneity,^13^ while Egger’s test was used to evaluate funnel plot asymmetry to assess whether smaller cohorts led to higher effect sizes (ie, ‘small-study effects’).^14^ The results were then corrected for multiple testing at a false discovery rate (FDR) of 0.05.^15^ For each outcome significant in meta-analysis, we provide a breakdown per working status category per cohort. Comparisons were made using the Χ^2^ test.

### Sensitivity analysis

As mentioned above, we have calculated change scores for psychological distress, alcohol, smoking and sleep. We have pursued further adjustment for baseline states (ie, pre-pandemic mental state score, number of drinks per week, cigarettes smoked per day and sleep hours per night, respectively).

As we have scored the COVID-19 variable as 1, either for strong personal suspicion of infection or a positive antigen or antibody test, self-reporting bias was a concern. Therefore, we ran a sensitivity analysis where only a positive antigen or antibody was scored as 1. As having a COVID-19 test was highly selective in May 2020 with health and social care workers being prioritised, we have run an additional sensitivity analysis where we included only participants who had been tested.

## Results

**Figure 1:**
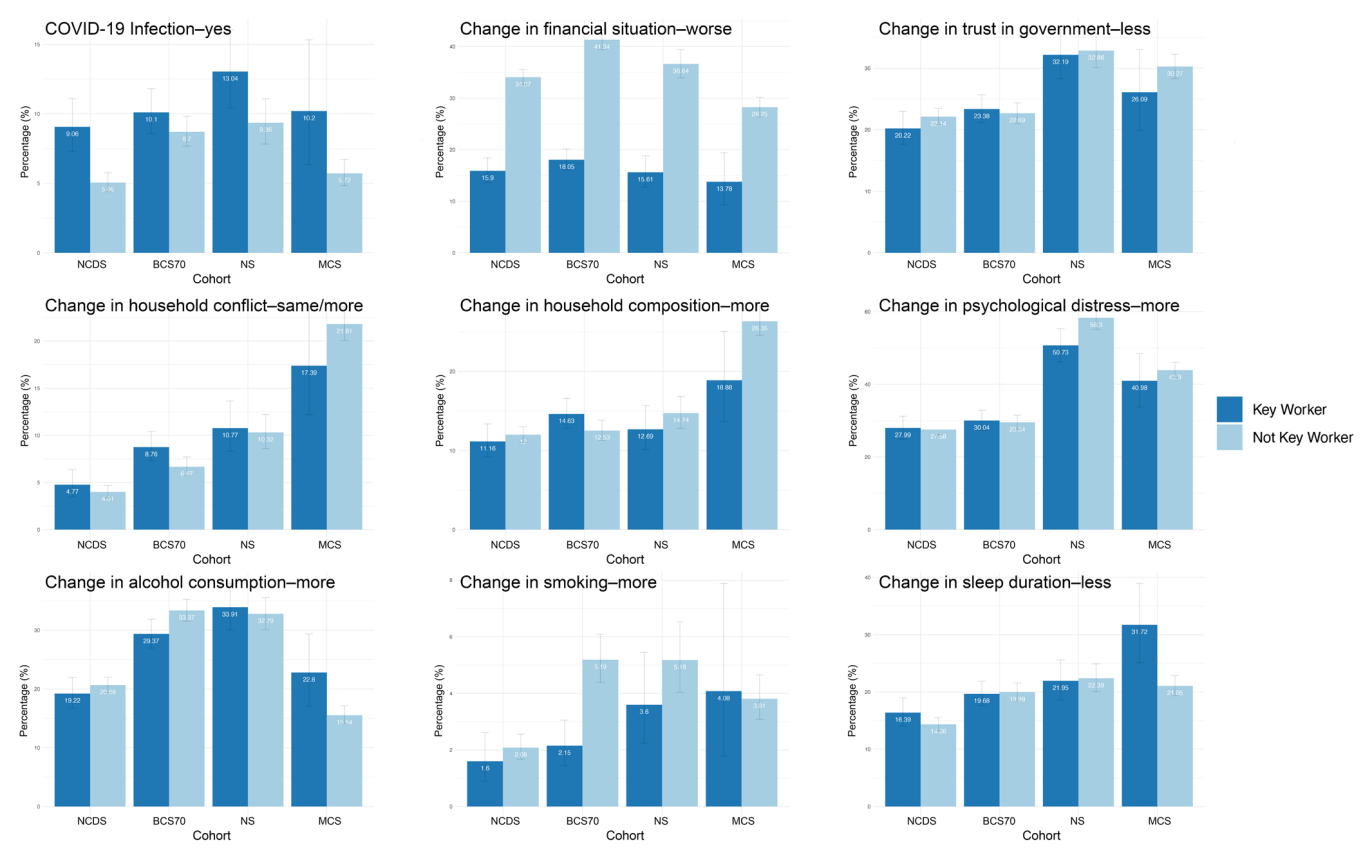
Bar charts illustrating the percentages of key workers versus non-key workers experiencing the various outcomes. Results are presented across the four UK longitudinal cohorts, ordered by decreasing age of the cohort from left to right. Error bars represent 95% CIs. BCS70, British Cohort Study; MCS, Millennium Cohort Study; NCDS, National Child Development Study; NS, Next Steps.

Overall, 13 953 out of 38 727 participants (36%) responded to the survey as follows: 5178 out of 8943 (58%) for NCDS, 4223 out of 10 458 for BCS70 (40%), 1907 out of 9380 (20%) for NS and 2645 out of 9946 (27%) for MCS. Responding to the survey was associated with being female, having a higher adult SEP and better self-rated health state. After removing all participants who lacked at least one outcome data, 13 736 participants were included in our quantitative analyses, 3113 of which were key workers (characteristics summarised in table 1). Comparisons of the outcome data based on key worker status are presented in figure 1. Regression results from the fully adjusted models are presented in table 2, while the results for intermediate models can be found in online supplemental table S2. For outcomes where we calculated change scores, results when adjustment for baseline state (over and above the covariates in the fully adjusted models) was pursued are presented in online supplemental table S3. Meta-analyses results are summarised in table 3.

**Table 1:** Characteristics of participants by cohort

Participant characteristics	Cohort study birth year			
	1958	1970	1989–1990	2000–2002
Sample size				
Questionnaire respondents (n=13 953)	5178	4223	1907	2645
Included participants (n=13 736)	5119	4132	1876	2609
Participant characteristics				
Age (years)	62	50	30–31	19–20
Males (%)	2432 (47.51)	1708 (41.48)	633 (34.76)	770 (29.51)
Non-white ethnicity (%)	N/A	N/A	361 (19.27)	367 (14.17)
Adult SEP GCSE—none (%)	1837 (36.61)	1416 (36.43)	434 (24.82)	986 (40.12)
Childhood SEP I–III (%)	1897 (43.61)	1727 (48.74)	1227 (69.36)	1755 (79.70)
Chronic Illness (%)	3099 (61.24)	1955 (48.08)	715 (39.20)	830 (33.33)
Shielding letter (%)	334 (6.57)	196 (4.77)	56 (3.00)	60 (2.30)
Key workers (%)	938 (18.32)	1396 (33.79)	583 (31.08)	196 (7.51)
Outcomes				
COVID-19 infection—yes (%)	296 (5.78)	379 (9.18)	197 (10.50)	158 (6.06)
Change in financial situation—worse (%)	1504 (30.61)	1331 (33.23)	534 (29.80)	658 (27.08)
Change in trust in government—less (%)	1035 (21.78)	889 (22.93)	550 (32.64)	680 (29.93)
Change in conflict—same/more (%)	197 (4.15)	287 (7.41)	177 (10.47)	488 (21.45)
Change in household composition—yes (%)	586 (11.84)	532 (13.25)	256 (14.08)	644 (25.76)
Change in psychological distress—more (%)	1215 (27.65)	989 (29.71)	825 (55.86)	1885 (43.70)
Change in alcohol consumption—more (%)	953 (20.38)	1235 (31.97)	580 (33.16)	380 (16.14)
Change in cigarette smoking—more (%)	102 (1.99)	172 (4.16)	88 (4.69)	100 (3.83)
Change in sleep duration—less (%)	708 (14.74)	777 (19.87)	384 (22.25)	511 (21.90)

The percentages have been derived after subtracting the number of missing data.

1958 refers to National Child Development Study; 1970 refers to British Cohort Study; 1989–1990 refers to Next Steps; 2000–2002 refers to Millennium Cohort Study.

GCSE, General Certificate of Secondary Education; N/A, not available; SEP, socioeconomic position.

**Table 2:** Association of key worker status with outcomes showing results for the fully adjusted model 3 only

Cohort	Effect size	COVID-19 infection*	Change in financial situation†	Change in trust in government‡	Change in conflict§	Change in household composition¶	Change in psychological distress**	Change in alcohol consumption††	Change in smoking‡‡	Change in sleep duration§§
1958	OR (95% CI)	1.95 (1.45 to 2.61)	0.34 (0.27 to 0.42)	0.94 (0.76 to 1.14)	1.16 (0.91 to 1.51)	0.92 (0.71 to 1.18)	1.00 (0.82 to 1.21)	0.91 (0.73 to 1.11)	0.78 (0.40 to 1.40)	1.16 (0.93 to 1.45)
	P value	**<0.0001**	**<0.0001**	0.516	0.244	0.522	0.990	0.344	0.428	0.184
1970	OR (95% CI)	1.18 (0.92 to 1.51)	0.31 (0.26 to 0.37)	1.12 (0.94 to 1.34)	1.38 (1.11 to 1.72)	1.17 (0.94 to 1.44)	1.04 (0.87 to 1.24)	0.77 (0.66 to 0.90)	0.43 (0.27 to 0.66)	0.95 (0.79 to 1.14)
	P value	0.184	**<0.0001**	0.191	**0.004**	0.160	0.669	**0.001**	**0.0002**	0.577
1989–1990	OR (95% CI)	1.36 (0.98 to 1.90)	0.29 (0.22 to 0.38)	0.95 (0.75 to 1.21)	1.00 (0.69 to 1.40)	0.87 (0.63 to 1.19)	0.77 (0.61 to 0.98)	1.01 (0.80 to 1.27)	0.70 (0.39 to 1.21)	0.94 (0.72 to 1.22)
	P value	0.067	**<0.0001**	0.695	0.994	0.388	**0.034**	0.941	0.214	0.651
2000–2002	OR (95% CI)	1.47 (0.78 to 2.76)	0.39 (0.24 to 0.65)	0.75 (0.49 to 1.11)	0.89 (0.56 to 1.37)	0.72 (0.46 to 1.07)	0.84 (0.59 to 1.20)	1.35 (0.87 to 2.04)	0.93 (0.32 to 2.20)	1.64 (1.11 to 2.38)
	P value	0.234	**0.0002**	0.158	0.614	0.118	0.340	0.172	0.895	**0.011**

All analyses used generalised linear models with logit link. Significant p values are highlighted in bold.

Adjustment was made for the individualised combined weight (accounting for non-response and stratified cohort design where appropriate), sex, ethnicity, highest educational attainment (adult SEP), childhood SEP, presence of chronic illness and shielding letter.

*COVID-19 infection was coded as 0=no, 1=yes.

†Change in financial situation was coded as 0=same or better, 1=worse.

‡Change in trust in government was coded as 0=same or more, 1=less.

§Change in conflict during lockdown was coded as 0=less, 1=same or more.

¶Change in household composition during lockdown was coded as 0=no, 1=yes.

**Change in psychological distress during lockdown was coded as 1=an increase in the mental health score, 0=same or lower score.

††Change in alcohol consumption was coded as 1=more drinks per week during lockdown compared with pre-lockdown or 0=same or lower.

‡‡Change in smoking was coded as 0=same or less cigarettes smoked during lockdown compared with before lockdown, 1=more cigarettes.

§§Change in sleep duration was coded as 0=same or more hours slept during lockdown and 1=less hours slept during lockdown.

SEP, socioeconomic position.

**Table 3:** Meta-analysis for the associations between being a key worker and outcomes

Outcome	n	Study heterogeneity			OR (95% CI)	P value	Egger’s test p value
		I^2^	Q	P value			
COVID-19 infection*	11 076	54.72%	6.63	0.085	1.43 (1.22 to 1.68)	**<0.0001**	0.801
Change in financial situation†	10 649	0.00%	1.73	0.631	0.32 (0.24 to 0.65)	**<0.0001**	0.534
Change in trust in government‡	10 236	29.57%	4.26	0.235	1.00 (0.89 to 1.11)	0.932	0.126
Change in conflict§	10 234	29.86%	4.28	0.233	1.19 (1.03 to 1.37)	**0.016**	0.137
Change in household composition¶	10 736	44.96%	5.45	0.142	0.98 (0.85 to 1.12)	0.743	0.073
Change in psychological distress**	9877	34.71%	4.60	0.204	0.95 (0.85 to 1.05)	0.320	0.293
Change in alcohol consumption††	10 269	62.46%	7.99	**0.046**	0.88 (0.79 to 0.98)	**0.022**	0.052
Change in smoking‡‡	11 076	24.09%	3.95	0.257	0.60 (0.44 to 0.80)	**0.005**	0.144
Change in sleep duration§§	10 371	61.75%	7.84	**0.049**	1.06 (0.94 to 1.19)	0.350	0.245

All analyses used a random-effects model meta-analysis. Significant p values which persisted at a false discovery rate of 0.05 are highlighted in bold.

*COVID-19 infection was coded as 0=no, 1=yes.

†Change in financial situation was coded as 0=same or better, 1=worse.

‡Change in trust in government was coded as 0=same or more, 1=less.

§Change in conflict during lockdown was coded as 0=less, 1=same or more.

¶Change in household composition during lockdown was coded as 0=no, 1=yes.

**Change in psychological distress during lockdown was coded as 1=an increase in the mental health score, 0=same or lower score.

††Change in alcohol consumption was coded as 1=more drinks per week during lockdown compared with pre-lockdown or 0=same or lower.

‡‡Change in smoking was coded as 0=same or less cigarettes smoked during lockdown compared with before lockdown, 1=more cigarettes.

§§Change in sleep duration was coded as 0=same or more hours slept during lockdown and 1=less hours slept during lockdown.

### COVID-19 infection during lockdown

COVID-19 infection was associated with key worker status in the NCDS cohort (OR 1.95, 95% CI 1.45 to 2.61, p<0.0001, table 2). The meta-analysis revealed that COVID-19 infection was associated with being a key worker across all cohorts with a pooled OR 1.43 (95% CI 1.22 to 1.68, p<0.0001) even at an FDR of 0.05. There was moderate heterogeneity between the cohorts (I^2^=52.72%, p=0.085, table 3).

Significant associations persisted when COVID-19 infection status was scored only based on a positive antigen or antibody test. The meta-analysis revealed a pooled OR 2.74 (95% CI 1.46 to 5.13, p=0.002) (online supplemental table S4). Across all samples, 333 individuals were tested (64 were still waiting their results and 8 had an inconclusive test). In the tested population only, significant associations did not persist. In general, key workers were more likely to have COVID-19 infection compared with other workers and those not working/retired (online supplemental table S5).

### Changes in financial situation during lockdown

Across all cohorts, key workers were less likely to be financially worse-off during lockdown with a pooled OR 0.32 (95% CI 0.24 to 0.65, p<0.0001) on the background of insignificant intercohort heterogeneity (I^2^=0.00%, p=0.631). The association persisted despite correction for multiple testing. Smaller cohorts were not associated with larger effect sizes (Egger’s test p value=0.090, table 3). In general, key workers were less likely to be financially worse-off compared with any other working status category (online supplemental table S5).

### Changes in trust in government during lockdown

Key worker status was not associated with changes in trust in government in any of the cohorts (table 2).

### Changes in conflict during lockdown

In the BCS70, being a key worker was associated with a change in conflict with the people around (OR 1.38, 95% CI 1.11 to 1.72, p=0.004). The meta-analysis revealed that key workers were more likely to experience same or more conflict during lockdown (OR 1.19, 95% CI 1.03 to 1.37, p=0.016) even at an FDR of 0.05. There was low heterogeneity between cohorts (I^2^=29.86%, p=0.233) (table 3).

### Changes in household composition during lockdown

Key worker status was not associated with changes in household composition in any of the cohorts (table 2).

### Changes in psychological distress during lockdown

In the NS cohort, key workers were less likely to have a higher GHQ-12 score (OR 0.77, 95% CI 0.61 to 0.98, p=0.034). However, the meta-analyses revealed that being a key worker was not associated with psychological distress (OR 0.95, 95% CI 0.85 to 1.05, p=0.320, table 2).

### Changes in alcohol consumption during lockdown

Only in the BCS70 cohort, key worker status was associated with drinking a lower number of alcoholic drinks per week compared with pre-lockdown (OR 0.77, 95% CI 0.66 to 0.90, p=0.001). The association remained significant even after adjusting for the number of drinks per week prior to lockdown (online supplemental table S3). The meta-analysis confirmed that the association was observed across all cohorts as the pooled OR was 0.88 (95% CI 0.79 to 0.98, p=0.022, table 3) even at an FDR of 0.05. The heterogeneity was high (I^2^=62.46%, p=0.046). Key workers were less likely to consume more alcohol compared with volunteers, those in teaching and non-working/retired people (online supplemental table S5).

### Changes in smoking during lockdown

In the BCS70 cohort, key workers were less likely to smoke more cigarettes (OR 0.43, 95% CI 0.27 to 0.66, p=0.002, table 2). Even after adjusting for the number of cigarettes smoked per day before lockdown, the association remained (online supplemental table S3).

The association persisted in the meta-analysis as well (pooled OR 0.60, 95% CI 0.44 to 0.80, p=0.005, table 3), on a background of a relatively low heterogeneity (I^2^=24.09%, p=0.257) and despite correction for multiple testing. Key workers were less likely to smoke more cigarettes compared with those not working/retired.

### Changes in sleep during lockdown

Only participants from the MCS cohort reported that they slept less since the UK national lockdown (OR 1.64, 95% CI 1.11 to 2.38, p=0.011). The association persisted even after adjusting for the number of hours slept before lockdown (online supplemental table S3).

However, the meta-analysis failed to confirm such association (pooled OR 1.06, 95% CI 0.94 to 1.19, p=0.350, table 3). Heterogeneity was high (I^2^=61.75%, p=0.049).

## Discussion

### Findings statement

Our data from the four UK national longitudinal studies during the COVID-19 lockdown (May 2020) show that key worker status was associated with both negative (more COVID-19 infection and conflict with people around) and positive outcomes (financial stability, less drinks per week and less cigarettes per day). There was no association between being a key worker and changes in household composition, trust in government, psychological distress or sleeping less during lockdown.

### Interpretation

Being a key worker during the UK national lockdown was a challenging experience. Key workers were three times less likely to be worse-off financially, potentially due to their job essentiality during the pandemic. Key workers do not appear to have experienced more psychological distress than the rest of the population based on our meta-analysis, although psychological distress in key workers has been previously reported towards the start of the pandemic.^3^ As lockdown progressed, businesses and the civil society began to manifest more publicly their deep appreciation of key workers’ efforts, through the weekly national ‘clap for carers’ and similar activities, thus it is plausible that these could have had a positive psychological impact on key workers. Although the media has reported isolated cases of key workers having to leave their homes in order to protect vulnerable household members, we did not observe an association with change in household composition in any of our cohorts. Instead, we found that for health behaviours that were to some extent within key workers’ control, these were all positive as they were less likely to drink or smoke more and did not report reduced sleep duration. The ability to remain in stable employment with serious job obligations at a time when most were either being furloughed or rendered redundant could have fuelled a sense of duty which translated into more responsible health behaviours. Lastly, the trust in the government was not affected. Speculatively, this might mean that key workers were still trusting the government’s ability to deal with the pandemic.

On the other hand, as many of the key workers were more exposed to COVID-19 than the rest of the population during lockdown, their infection rates were higher. As this risk could have been avoided by better provision of personal protective equipment and implementation of firmer social distancing measures to protect staff, the government needs to ensure that better protection is provided for the ongoing third wave. In addition, the UK COVID-19 vaccines delivery plan should ensure better access to the vaccine programme for non-healthcare key workers as well. Key workers tended to experience more conflict with the people around them, some of which might have been augmented by relatives’ fear of getting infected, or key workers’ worries over bringing the virus home to their loved ones.

### Implications for key stakeholders

Pandemics can last multiple years and new viral mutations can arise.^16^ With the worrying emergence of multiple new COVID-19 strains, some of which could potentially be vaccine resistant,^17^ concerns about the prospect of a fourth wave and a fourth lockdown are more than justified.

UK policymakers know about the higher risk of COVID-19 infections in key workers, and they have a duty to implement better protective measures to those used in the first wave, which may not have been enough. Potential solutions include the free and more abundant supply of personal protective equipment, the free provision of COVID-19 rapid lateral flow tests and the enforcement of more secure work-related controls to ensure that the employers are complying with the COVID-19 legislation and are protecting the well-being of their key workers. Although we did not directly explore the impact of lockdown on the household members of key workers, the excess conflict is a worrying finding and suggests that the government should do more to support the families of key workers as a whole, including through support groups and tangible benefits where appropriate.

### Strengths and weaknesses of the study

The main strengths of the study are the large number of participants included, age-matching within the cohorts by design and cohort data spanning multiple generations from 19 to 74 years old. The longitudinal nature of the cohorts enabled the derivation of non-response weights which have been included in all analyses to address the data missingness issue.

Limitations include low response rates (especially in younger cohorts such as NS and MCS). Data binarisation enabled us to generate singular ORs across the cohorts to be used in meta-analysis, but continuous non-linear effects would be missed. Except for the psychological distress, which was completely based on validated scores, the majority of survey questions employed to derive our outcome variables have not been previously externally validated. COVID-19 infection status included personal suspicion of disease as mass testing was not yet nationally available at the time. This could have led to reporting bias; however, the sensitivity meta-analysis showed that indeed laboratory-confirmed COVID-19 diagnosis was also associated with key worker status. As the change in financial situation, trust in government, conflict and household composition were self-rated, they are prone to perception bias. The change in household composition and change in conflict variables do not take into account individuals who might be living in isolation, although we have adjusted for the receipt of a shielding letter. We did not record key workers according to the Standard Occupational Classification and the Standard Industrial Classification of the Economic Activities, did not capture the type of key workers and thus did not distinguish between frontline workers from others potentially underestimating the burden of lockdown in healthcare workers. As the prevalence of outcomes varied across the cohorts, the OR interpretation for cohort comparison is subjected to bias. Meta-analyses with high heterogeneity may largely reflect age group differences as the potential overall between the cohort samples is all. However, we were unable to formally test whether age cohort as a continuous moderator is a source of heterogeneity. The associations between our outcomes and age are probably non-linear, but non-linear meta-regression (eg, using cubic polynomial or splines) would be inappropriate with four data points. On a background of high heterogeneity, the potential of having spurious pooled OR is a possibility. Lastly, performing extensive analyses to separate pandemic effects from known confounders such as seasonal variation was beyond the scope of our study.

### Future directions

Further studies are required to assess the multidimensional impact of the COVID-19 pandemic on key workers and their family members. Public health measures implemented to protect key workers should be under continuous review and subject to constant national audits to ensure they are effective and readily accessible.

## Conclusion

Being a key worker at the height of the UK national lockdown was a double-edged sword. On one hand, key workers had financial stability and made better lifestyle choices compared with all non-key workers. On the other hand, they were more likely to contract COVID-19 and experience conflict. Despite multiple media reports claiming the contrary, being a key worker was not associated with a change in psychological distress. The UK government had the basic duty to protect its key workers from SARS-CoV-2 infection, but it may have failed to do so, and there is an urgent need to rectify this in light of the ongoing third wave.

**What is already known on this subject**

- Key workers played a monumental role in the UK’s response to the COVID-19 pandemic especially during lockdown. The UK government has taken multiple measures to protect the key workers. However, the impact of the lockdown on key workers is still mostly unknown.

**What this study adds**

- Our findings show that key workers were more likely to report COVID-19 symptoms or have a COVID-19-positive antibody/antigen test. In addition, they were more likely to experience conflict. However, they were less likely to consume more alcohol or smoke more. Lastly, key worker status was not associated with psychological distress, changes in household composition or trust in the government during lockdown.

## Data Availability

Data are available upon reasonable request. Data from the cohorts are available from the UK data archive: https://www.data-archive.ac.uk.

## References

[1] The Lancet. The plight of essential workers during the COVID-19 pandemic. Lancet 2020;395:1587. 10.1016/S0140-6736(20)31200-9 32446399 PMC7241973

[2] Greenberg N, Docherty M, Gnanapragasam S, et al. Managing mental health challenges faced by healthcare workers during covid-19 pandemic. BMJ 2020;368:m1211. 10.1136/bmj.m1211 32217624

[3] Pierce M, Hope H, Ford T, et al. Mental health before and during the COVID-19 pandemic: a longitudinal probability sample survey of the UK population. Lancet Psychiatry 2020;7:883–92. 10.1016/S2215-0366(20)30308-4 32707037 PMC7373389

[4] The Lancet. COVID-19: protecting health-care workers. Lancet 2020;395:922. 10.1016/S0140-6736(20)30644-9 PMC7138074 32199474

[5] Power C, Elliott J. Cohort profile: 1958 British birth cohort (National child development study). Int J Epidemiol 2006;35:34–41. 10.1093/ije/dyi183 16155052

[6] Elliott J, Shepherd P. Cohort profile: 1970 British birth cohort (BCS70). Int J Epidemiol 2006;35:836–43. 10.1093/ije/dyl174 16931528

[7] Connelly R, Platt L. Cohort profile: UK millennium cohort study (mcs). Int J Epidemiol 2014;43:1719–25. 10.1093/ije/dyu001 24550246

[8] Calderwood L, Sanchez C. Next steps (formerly known as the longitudinal study of young people in England). Open Health Data 2016;4:e2–e. 10.5334/ohd.16

[9] Montazeri A, Harirchi AM, Shariati M, et al. The 12-Item general health questionnaire (GHQ-12): translation and validation study of the Iranian version. Health Qual Life Outcomes 2003;1:66. 10.1186/1477-7525-1-66 14614778 PMC280704

[10] Bowling A, Pikhartova J, Dodgeon B. Is mid-life social participation associated with cognitive function at age 50? results from the British National child development study (NCDS). BMC Psychol 2016;4:2016. 10.1186/s40359-016-0164-x PMC5134123 27908287

[11] Rodgers B, Pickles A, Power C, et al. Validity of the malaise inventory in general population samples. Soc Psychiatry Psychiatr Epidemiol 1999;34:333–41. 10.1007/s001270050153 10422488

[12] Kessler RC, Green JG, Gruber MJ, et al. Screening for serious mental illness in the general population with the K6 screening scale: results from the who world mental health (WMH) survey initiative. Int J Methods Psychiatr Res 2010;19:4–22. 10.1002/mpr.310 20527002 PMC3659799

[13] Higgins JPT, Thompson SG, Deeks JJ, et al. Measuring inconsistency in meta-analyses. BMJ 2003;327:557–60. 10.1136/bmj.327.7414.557 12958120 PMC192859

[14] Egger M, Davey Smith G, Schneider M, et al. Bias in meta-analysis detected by a simple, graphical test. BMJ 1997;315:629–34. 10.1136/bmj.315.7109.629 9310563 PMC2127453

[15] Benjamini Y, Hochberg Y. Controlling the false discovery rate: a practical and powerful approach to multiple testing. J R Stat Soc Series B 1995;57:289–300. 10.1111/j.2517-6161.1995.tb02031.x

[16] He D, Dushoff J, Day T, et al. Inferring the causes of the three waves of the 1918 influenza pandemic in England and Wales. Proc Biol Sci 2013;280:20131345. 10.1098/rspb.2013.1345 23843396 PMC3730600

[17] Kennedy DA, Read AF. Monitor for COVID-19 vaccine resistance evolution during clinical trials. PLoS Biol 2020;18:e3001000–e. 10.1371/journal.pbio.3001000 33166303 PMC7676675

