# Supplementary material for: Impact of lockdown on key workers: findings from the COVID-19 survey in four UK national longitudinal studies
Source: J Epidemiol Community Health. 2021 Apr 9:jech-2020-215889. doi: 10.1136/jech-2020-215889 (PMC8042596; doi:10.1136/jech-2020-215889)
Supplement: Supplementary file 1 [file jech-2020-215889supp001.pdf]

SUPPLEMENTARY MATERIAL FOR:

Impact of lockdown on keyworkers – Findings from the COVID-19 survey in four UK national longitudinal studies

Constantin-Cristian Topriceanu[1], Andrew Wong[1], James C Moon[2,3], Alun D Hughes[1], Nish Chaturvedi[1], Gabriella Conti[4], David Bann[5], Praveetha Patalay[1,5], Gabriella Captur[1,2,6]

Author Affiliations:
1. UCL MRC Unit for Lifelong Health and Ageing, University College London, London WC1E 7HB
2. UCL Institute of Cardiovascular Science, University College London, Gower Street, London WC1E 6BT, UK
3. Cardiac MRI Unit, Barts Heart Centre, West Smithfield, London, EC1A 7BE
4. Department of Economics and UCL Social Research Institute, University College London, London WC1 H0AX, UK
5. Centre for Longitudinal Studies, Social Research Institute, University College London, London WC1H 0AL, UK
6. The Royal Free Hospital, Centre for Inherited Heart Muscle Conditions, Cardiology Department, Pond Street, Hampstead, London NW3 2QG, UK

Supplementary Table S1. Survey questions.

**Survey Derived Variable**[*]	**Survey Question**
**Exposure**	
Key Worker	Are you a Key worker, or has your work been classified as critical to the Covid-19 response?
**Covariates**	
Chronic illness	Do you have any of the following? Please select all that apply:<br>CM suffering from a long-standing illness
Shielding Letter	Have you received a letter or text message from the NHS or Chief Medical Officer saying that you have been identified as someone at risk of severe illness if you catch Coronavirus, because you have an underlying disease or health condition?
**Outcomes**	
COVID-19	Do you think that you have or have had Coronavirus?<br>Have you been tested for Coronavirus?<br>What was the result of your coronavirus test? If you had more than one test please report the findings of the latest test.
Change in financial situation	Overall, how do you feel your current financial situation compares to before the Coronavirus outbreak?
Change in trust in government	Since the Coronavirus outbreak please indicate how the following have changed. The amount of trust I have in the Government
Change in conflict	Since the Coronavirus outbreak please indicate how the following have changed. The amount of conflict I have had with people around me
Change in household composition	Have there been any changes to the people you are living with since the Coronavirus outbreak?
Change in psychological distress	**1958 and 1970 cohorts only**<br>The next questions are concerned with how you are feeling generally. Do you feel tired most of the time?<br>The next questions are concerned with how you are feeling generally. Do you often feel miserable or depressed?<br>The next questions are concerned with how you are feeling generally. Do you often get worried about things?<br>The next questions are concerned with how you are feeling generally. Do you often get in a violent rage?<br>The next questions are concerned with how you are feeling generally. Do you often suddenly become scared for no good reason?<br>The next questions are concerned with how you are feeling generally. Are you easily upset or irritated?<br>The next questions are concerned with how you are feeling generally. Are you constantly keyed up and jittery?<br>The next questions are concerned with how you are feeling generally. Does every little thing get on your nerves and wear you out?<br>The next questions are concerned with how you are feeling generally. Does your heart often race like mad?<br><br>**1989-1990 cohort only**<br>Have you recently been able to concentrate on what you’re doing?<br>Have you recently lost much sleep over worry?<br>Have you recently felt that you are playing a useful part in things?<br>Have you recently felt capable of making decisions about things?

	Have you recently felt constantly under strain?<br>Have you recently felt you couldn't overcome your difficulties?<br>Have you recently been able to enjoy your normal day to day activities?<br>Have you recently been able to face up to your problems?<br>Have you recently been feeling unhappy or depressed?<br>Have you recently been losing confidence in yourself?<br>Have you recently been thinking of yourself as a worthless person?<br>Have you recently been feeling reasonably happy, all things considered?<br><br>**2000-2002 cohort only**<br>The next few questions are about how you have felt over the last 30 days. During the last 30 days, about how often did you feel so depressed that nothing could cheer you up?<br>During the last 30 days, about how often did you feel hopeless?<br>During the last 30 days, about how often did you feel restless or fidgety?<br>During the last 30 days, about how often did you feel that everything was an effort?<br>During the last 30 days, about how often did you feel worthless?<br>During the last 30 days, about how often did you feel nervous?
Change in alcohol consumption	In the month before the Coronavirus outbreak, how many standard alcoholic drinks have you had on a typical day when you were drinking?<br>In the month before the Coronavirus outbreak, how often did you have a drink containing alcohol?<br>Since the start of the Coronavirus outbreak, how many standard alcoholic drinks do you have on a typical day when you were drinking?<br>Since the start of the Coronavirus outbreak, how often have you had a drink containing alcohol?
Change in cigarette smoking	In the month before the Coronavirus outbreak, how many cigarettes a day did you usually smoke?<br>Since the start of the Coronavirus outbreak, how many cigarettes a day have you typically smoked?
Change in sleep duration	In the month before the start of the Coronavirus outbreak, how many hours did you sleep each night on average?<br>Since the start of the Coronavirus outbreak, how many hours have you slept each night on average?

* Any other variable used was derived from the cohorts' longitudinal data as described in the methods section of the manuscript.
1958 refers to National Child Development Study (NCDS); 1970 refers to British Cohort Study (BCS70); 1989-1990 refers to Next Steps (NS); 2000-2002 refers to Millennium Cohort Study (MCS); SEP, socio-economic position; GCSE, general certificate of secondary education; N/A, not available; yrs, years.

Supplementary Table S2. Association of key worker status with outcomes.

Outcome	Cohort study birth year	Model 1 ^		Model 2 *		Model 3†	
		**OR (95% CI)**	***p*-value**	**OR (95% CI)**	***p*-value**	**OR (95% CI)**	***p*-value**
**COVID-19 infection[a]**	**1958**	1.87 (1.43, 2.43)	**<0.0001**	1.94 (1.45, 2.59)	**<0.0001**	1.95 (1.45, 2.61)	**<0.0001**
	**1970**	1.20 (0.96, 1.50)	0.100	1.21 (0.95, 1.55)	0.118	1.18 (0.92, 1.51)	0.184
	**1989-1990**	1.44 (1.06, 1.96)	**0.020**	1.42 (1.02, 1.96)	**0.039**	1.36 (0.98, 1.90)	0.067
	**2000-2002**	1.89 (1.12, 3.03)	**0.012**	1.57 (0.84, 2.74)	0.132	1.47 (0.75, 2.65)	0.234
**Change in financial situation[b]**	**1958**	0.37 (0.31, 0.44)	**<0.0001**	0.35 (0.28, 0.43)	**<0.0001**	0.34 (0.27, 0.42)	**<0.0001**
	**1970**	0.31 (0.26, 0.36)	**<0.0001**	0.31 (0.26, 0.37)	**<0.0001**	0.31 (0.26, 0.36)	**<0.0001**
	**1989-1990**	0.31 (0.24, 0.40)	**<0.0001**	0.29 (0.22, 0.38)	**<0.0001**	0.29 (0.22, 0.38)	**<0.0001**
	**2000-2002**	0.41 (0.26, 0.61)	**<0.0001**	0.40 (0.24, 0.63)	**0.0002**	0.39 (0.23, 0.62)	**0.0002**
**Change in trust in government[c]**	**1958**	0.90 (0.75, 1.07)	0.238	0.94 (0.77, 1.15)	0.565	0.94 (0.76, 1.14)	0.516
	**1970**	1.05 (0.90, 1.23)	0.559	1.09 (0.92, 1.30)	0.304	1.12 (0.94, 1.34)	0.191
	**1989-1990**	0.99 (0.79, 1.23)	0.922	0.95 (0.75, 1.21)	0.687	0.95 (0.75, 1.21)	0.695
	**2000-2002**	0.85 (0.60, 1.19)	0.348	0.75 (0.50, 1.11)	0.160	0.75 (0.49, 1.11)	0.158
**Change in conflict[d]**	**1958**	1.09 (0.88, 1.37)	0.432	1.13 (0.89, 1.46)	0.336	1.16 (0.91, 1.51)	0.244
	**1970**	1.43 (1.18, 1.75)	**0.0003**	1.35 (1.09, 1.67)	**0.006**	1.38 (1.11, 1.72)	**0.004**
	**1989-1990**	1.08 (0.77, 1.50)	0.648	1.00 (0.69, 1.40)	0.900	1.00 (0.69, 1.43)	0.994
	**2000-2002**	0.76 (0.50, 1.11)	0.165	0.85 (0.54, 1.30)	0.464	0.89 (0.56, 1.37)	0.613
**Change in household composition[e]**	**1958**	0.91 (0.73, 1.14)	0.439	0.93 (0.72, 1.19)	0.589	0.92 (0.71, 1.18)	0.522
	**1970**	1.20 (0.99, 1.45)	0.058	1.20 (0.97, 1.48)	0.094	1.17 (0.94, 1.44)	0.160
	**1989-1990**	0.83 (0.62, 1.11)	0.221	0.87 (0.63, 1.19)	0.378	0.87 (0.63, 1.19)	0.388
	**2000-2002**	0.62 (0.42, 0.89)	**0.012**	0.74 (0.49, 1.10)	0.145	0.72 (0.46, 1.07)	0.118

**Change in psychological distress[f]**	**1958**	1.02 (0.86, 1.21)	0.801	1.00 (0.83, 1.21)	0.991	1.00 (0.82, 1.21)	0.990
	**1970**	1.03 (0.88, 1.20)	0.725	1.06 (0.89, 1.25)	0.529	1.04 (0.87, 1.24)	0.669
	**1989-1990**	0.73 (0.59, 0.92)	**0.006**	0.76 (0.60, 0.97)	**0.025**	0.77 (0.61, 0.98)	**0.034**
	**2000-2002**	0.88 (0.64, 1.19)	0.410	0.85 (0.60, 1.20)	0.356	0.84 (0.59, 1.20)	0.340
**Change in alcohol consumption[g]**	**1958**	0.90 (0.75, 1.09)	0.284	0.91 (0.74, 1.12)	0.379	0.91 (0.73, 1.11)	0.344
	**1970**	0.81 (0.70, 0.94)	**0.005**	0.76 (0.65, 0.89)	**0.0008**	0.77 (0.66, 0.90)	**0.001**
	**1989-1990**	1.01 (0.82, 1.25)	0.909	1.00 (0.80, 1.26)	0.981	1.01 (0.80, 1.27)	0.941
	**2000-2002**	1.55 (1.07, 1.46)	**0.172**	1.43 (0.94, 2.14)	0.086	1.35 (0.87, 2.04)	0.172
**Change in smoking[h]**	**1958**	0.77 (0.42, 1.29)	0.348	0.77 (0.39, 1.37)	0.399	0.78 (0.40, 1.40)	0.428
	**1970**	0.42 (0.28, 0.62)	**<0.0001**	0.42 (0.26, 0.64)	**0.0001**	0.43 (0.27, 0.66)	**0.002**
	**1989-1990**	0.63 (0.37, 1.04)	0.079	0.71 (0.40, 1.22)	0.235	0.70 (0.39, 1.21)	0.244
	**2000-2002**	1.02 (0.45, 2.02)	0.961	1.13 (0.43, 2.49)	0.776	0.93 (0.32, 2.17)	0.871
**Change in sleep duration[i]**	**1958**	1.15 (0.94, 1.40)	0.174	1.18 (0.94, 1.46)	0.145	1.16 (0.93, 1.45)	0.184
	**1970**	0.97 (0.82, 1.14)	0.675	0.94 (0.78, 1.13)	0.498	0.95 (0.79, 1.14)	0.577
	**1989-1990**	1.00 (0.78, 1.27)	0.983	0.95 (0.73, 1.23)	0.707	0.94 (0.72, 1.22)	0.651
	**2000-2002**	1.76 (1.26, 2.43)	**0.0008**	1.54 (1.05, 2.23)	**0.025**	1.64 (1.11, 2.38)	**0.011**

All analyses used generalized linear models with logit link. Significant *p*-values are highlighted in bold.
[a] COVID-19 infection was coded as 0=No, 1=Yes.
[b] Change in financial situation was coded as 0=Same or Better, 1=Worse.
[c] Change in trust in government was coded as 0=Same or more, 1=Less.
[d] Change in conflict during lockdown was coded as 0=Less, 1=Same or more.
[e] Change in household composition during lockdown was coded as 0=No, 1=Yes.
[f] Change in psychological distress during lockdown was coded as 1=an increase in the mental health score, 0=same or lower score.
[g] Change in alcohol consumption was coded as 1=more drinks per week during lockdown compared to pre-lockdown or 0=same or lower.
[h] Change in smoking was coded as 0=Same or less cigarettes smoked during lockdown compared to before lockdown, 1=More cigarettes.
[i] Change in sleep duration was coded as 0=same or more hours slept during lockdown and 1=less hours slept during lockdown.
^ Model 1 was adjusted for combined weight, sex and ethnicity.
* Model 2 was adjusted for combined weight, sex, ethnicity, highest educational attainment (adult SEP) and childhood SEP.
[†] Model 3 was adjusted for combined weight, sex, ethnicity, highest educational attainment (adult SEP) childhood SEP, presence of chronic illness and shielding 1etter.
OR, odds ratio; CI, confidence interval.

Supplementary Table S3. Association of key worker status with outcomes showing results for the fully adjusted model 3 only.

Cohort	Effect size	Change in financial situation	Change in psychological distress	Change in alcohol consumption	Change in smoking	Change in sleep duration
**1958**	**OR** (95% CI)	0.34 (0.27, 0.42)	0.99 (0.81, 1.20)	0.89 (0.72, 1.09)	0.78 (0.40, 1.40)	1.18 (0.94, 1.47)
	***p*-value**	**<0.0001**	0.883	0.274	0.428	0.154
**1970**	**OR** (95% CI)	0.31 (0.26, 0.37)	1.02 (0.86, 1.22)	0.77 (0.65, 0.90)	0.39 (0.23, 0.65)	0.95 (0.79, 1.15)
	***p*-value**	**<0.0001**	0.825	**0.001**	**0.0004**	0.608
**1989-1990**	**OR** (95% CI)	0.30 (0.22, 0.39)	0.71 (0.55, 0.95)	1.04 (0.83, 1.31)	0.62 (0.31, 1.21)	0.96 (0.73, 1.25)
	***p*-value**	**<0.0001**	**0.021**	0.724	0.166	0.754
**2000-2002**	**OR** (95% CI)	0.39 (0.23, 0.62)	0.78 (0.51, 1.21)	1.30 (0.83, 1.98)	0.48 (0.15, 1.30)	1.78 (1.19, 2.61)
	***p*-value**	**0.0001**	0.274	0.229	0.178	**0.004**

All analyses used generalized linear models with logit link. Adjustment was made for combined weight, sex, ethnicity, highest educational attainment (adult SEP), childhood SEP, presence of chronic illness and shielding letter. All models for the psychological distress analyses have been further adjusted for psychological distress prior lockdown. All models for the alcohol analyses have been further adjusted for the number of drinks per week before the lockdown. All models for the sleep analyses have been further adjusted for the number of hours slept before the lockdown.

Abbreviations and coding as in ***Supplementary Table S2.*** Significant *p*-values are highlighted in bold.

Supplementary Table S4. Sensitivity analysis results for the COVID-19 infection outcome.

**Cohort**	**Effect size**	**Key worker COVID-19 infection: self-reported or positive test**	**Key worker COVID-19 infection: positive test only (whole sample)**	**Key worker COVID-19 infection: positive test only (only tested)**
**1958**	**OR (95% CI)**	1.95 (1.45, 2.61)	3.87 (1.52, 9.73)	2.97 (0.65, 17.10)
	***p*-value**	**<0.0001**	**0.004**	0.184
	n	296	19	19
**1970**	**OR (95% CI)**	1.18 (0.92, 1.51)	1.43 (0.42, 4.54)	0.39 (0.07, 1.92)
	***p*-value**	0.184	0.547	0.262
	n	379	17	17
**1989-1990**	**OR (95% CI)**	1.36 (0.98, 1.90)	2.81 (0.73, 11.50)	0.91 (0.13, 5.63)
	***p*-value**	0.067	0.129	0.923
	n	197	12	12
**2000-2002**	**OR (95% CI)**	1.47 (0.75, 2.65)	3.69 (0.17, 30.15)	0.54 (0.87, 1.48)
	***p*-value**	0.234	0.267	0.729
	n	158	7	7
**Pooled Results**	**OR (95% CI)**	1.43 (1.22, 1.68)	2.74 (1.46, 5.13)	1.00 (0.38, 2.61)
	***p*-value**	**<0.0001**	**0.002**	0.998
	n	1030	55	55

All analyses used generalized linear models with logit link. Significant *p*-values are highlighted in bold.
*Abbreviations and coding as in **Supplementary Table S2.***

Supplementary Table S5. Working status breakdown for outcomes significant in the meta-analysis.

Outcome	Cohort study birth year	Key workers[1]	Other workers[2]	*p*-value*	Volunteers[3]	*p*-value°	In teaching[4]	*p*-value^	Not working or retired[5]	*p*-value^{-}
**COVID-19 infection**	**1958**	85/853	58/955	**0.006**	13/327	**0.003**	7/145	0.094	133/2542	**<0.0001**
	**1970**	141/1255	101/1294	**0.009**	14/120	1.00	17/114	0.377	106/969	0.897
	**1989-1990**	76/507	61/561	0.094	4/40	0.602	9/96	0.263	47/475	**0.042**
	**2000-2002**	20/176	22/402	**0.033**	9/149	0.179	68/950	0.111	39/822	**0.003**
**Change in financial situation**	**1958**	149/788	326/657	**0.006**	113/224	**0.003**	56/93	0.094	860/1648	**<0.0001**
	**1970**	252/1144	417/956	**<0.0001**	72/58	**<0.0001**	71/57	**<0.0001**	519/460	**<0.0001**
	**1989-1990**	91/492	127/481	**0.023**	29/14	**<0.0001**	53/48	**<0.0001**	234/223	**<0.0001**
	**2000-2002**	27/169	70/326	0.276	43/64	**<0.0001**	241/751	**0.002**	277/462	**<0.0001**
**Change in household conflict**	**1958**	43/859	37/902	0.450	12/317	0.493	3/145	0.196	102/2325	0.549
	**1970**	110/1230	72/1258	**0.001**	14/114	0.508	11/114	1.00	71/864	0.357
	**1989-1990**	60/497	57/517	0.714	3/40	0.600	7/91	0.362	50/369	0.641
	**2000-2002**	32/152	65/299	0.989	27/78	0.124	224/717	0.072	140/541	0.395
**Change in alcohol consumption**	**1958**	172/723	206/727	**<0.0001**	70/251	**<0.0001**	35/110	**<0.0001**	470/1912	**<0.0001**
	**1970**	398/957	429/892	0.090	52/77	**0.013**	44/75	0.103	312/627	**0.045**
	**1989-1990**	195/380	199/392	0.980	17/26	0.560	33/67	0.949	136/304	0.345
	**2000-2002**	44/149	80/295	0.770	19/88	0.380	112/859	**<0.0001**	125/584	0.127
**Change in smoking**	**1958**	15/923	14/999	0.835	1/339	0.117	3/149	1.00	69/2607	0.113
	**1970**	30/1366	59/1336	**0.003**	11/123	**0.0001**	6/125	0.146	66/1010	**<0.0001**
	**1989-1990**	21/562	26/596	0.712	4/40	0.163	3/102	0.925	34/488	**0.037**
	**2000-2002**	8/188	21/403	0.785	9/101	0.214	19/999	0.097	43/818	0.724

For each working status category, results are reported as the counts of "1" responses and counts of "0" responses for each outcome category, separated by '/'. *p*-values are from Chi squared test. Significant *p*-values are highlighted in bold.

[1] Key worker status was self-assigned based on whether the participant reported that their work had been classified as critical to the COVID-19 response.
[2] In paid employment, but not key worker.
[3] In unpaid employment/volunteering.
[4] Not in paid or unpaid employment but receiving teaching during lockdown.
[5] Not working or retired.
* *p*-value for the comparison between key workers and other workers.
º *p*-value for the comparison between key workers and volunteers.
^ *p*-value for the comparison between key workers and those in teaching.
¯ *p*-value for the comparison between key workers and those not working/retired.

Other abbreviations and coding as in ***Supplementary Table S2.***
